# Nicotinamide adenine dinucleotide supplementation drives gut microbiota variation in Alzheimer’s mouse model

**DOI:** 10.3389/fnagi.2022.993615

**Published:** 2022-09-15

**Authors:** Xixia Chu, Yujun Hou, Qiong Meng, Deborah L. Croteau, Yong Wei, Supriyo De, Kevin G. Becker, Vilhelm A. Bohr

**Affiliations:** ^1^DNA Repair Section, National Institute on Aging, Baltimore, MD, United States; ^2^Institute for Regenerative Medicine, Shanghai East Hospital, Shanghai Key Laboratory of Signaling and Disease Research, Frontier Science Center for Stem Cell Research, School of Life Sciences and Technology, Tongji University, Shanghai, China; ^3^Laboratory of Genetics and Genomics, National Institute on Aging, Baltimore, MD, United States

**Keywords:** Alzheimer’s disease, nicotinamide riboside, gut microbiome, APP/PS1 transgenic mice, neuroinflammation, aging

## Abstract

Alzheimer’s disease (AD) is the most common neurodegenerative disease. Growing evidence suggests an important role for gut dysbiosis and gut microbiota-host interactions in aging and neurodegeneration. Our previous works have demonstrated that supplementation with the nicotinamide adenine dinucleotide (NAD^+^) precursor, nicotinamide riboside (NR), reduced the brain features of AD, including neuroinflammation, deoxyribonucleic acid (DNA) damage, synaptic dysfunction, and cognitive impairment. However, the impact of NR administration on the intestinal microbiota of AD remains unknown. In this study, we investigated the relationship between gut microbiota and NR treatment in APP/PS1 transgenic (AD) mice. Compared with wild type (WT) mice, the gut microbiota diversity in AD mice was lower and the microbiota composition and enterotype were significantly different. Moreover, there were gender differences in gut microbiome between female and male AD mice. After supplementation with NR for 8 weeks, the decreased diversity and perturbated microbial compositions were normalized in AD mice. This included the species *Oscillospira*, *Butyricicoccus*, *Desulfovibrio*, *Bifidobacterium*, *Olsenella*, *Adlercreutzia*, *Bacteroides*, *Akkermansia*, and *Lactobacillus*. Our results indicate an interplay between NR and host-microbiota in APP/PS1 mice, suggesting that the effect of NR on gut dysbiosis may be an important component in its therapeutic functions in AD.

## Introduction

Alzheimer’s disease (AD) is a progressive neurodegenerative disease and the most common cause of irreversible dementia among older individuals (Mattson, 2004). The two major hallmarks are amyloid beta (Aβ) plaques and hyperphosphorylated tau neurofibrillary tangles (Bloom, 2014). However, the pathology underlying the causality of these hallmarks and other AD features, including neuroinflammation, DNA damage, neurodegeneration, and dementia remain largely unknown. Currently, there is no cure for AD, and the approved therapies provide only temporary symptomatic relief (Athar et al., 2021).

The gut composition of mice is similar to humans at the phylum (90%) and genus (89%) levels, supporting the use of mice as a model organisms to study the gut microbiome (Krych et al., 2013). In recent years, it has been reported that gut microbiota play a critical role in AD pathology (Kowalski and Mulak, 2019). Perturbation of microorganisms in the gastrointestinal tract has been associated with aging, neurological and psychological disorders, including AD, autism spectrum disorder (ASD), multiple sclerosis (MS), Parkinson’s disease (PD), depression, and schizophrenia (Foster and McVey Neufeld, 2013; Dinan and Cryan, 2017; Vuong and Hsiao, 2017; Kelly et al., 2021; Tan et al., 2021). A previous study showed that the microbiota diversity decline in AD patients was accompanied by a decrease in the number of *Firmicutes* and *Actinobacteria* and an increase in *Bacteroidetes* and *Proteobacteria* at the phylum level (Vogt et al., 2017). Similar changes were observed in 8-month-old AD mice, with a decline of *Firmicutes*, *Verrucomicrobia*, and *Actinobacteria* and an increase of *Bacteroidetes* and *Tenericutes* compared to wild type mice (Harach et al., 2017). The alteration of gut microbiota is associated with aging, cognitive frailty and AD, demonstrating that the changes in the taxonomic composition of the intestinal flora might influence brain functions (Mariat et al., 2009; Ticinesi et al., 2018). It has been suggested that microbiome-derived functional amyloid and lipopolysaccharides (LPSs) may contribute to Aβ aggregation and neuroinflammation (Zhao et al., 2015; Pistollato et al., 2016). Interestingly, Aβ is also known as a natural antibiotic participating in the innate immune response, suggesting dual roles for Aβ in response to inflammation (Kumar et al., 2016). In addition, reduced Aβ deposition, accompanied by reduced neuroinflammation and increased Aβ degrading enzyme expression, were found in the brains of a germ-free APP/PS1 transgenic mice, implicating potential roles of gut bacteria on the development of Aβ (Harach et al., 2017). Furthermore, neuroinflammation is a major risk factor for aging and AD *via* activated microglia, the “brain’s resident immune cells” (Heneka et al., 2015). It has been demonstrated that host microbiota could regulate microglial homeostasis, suggesting an essential role of the brain-gut-microbiota axis in the pathogenesis of aging and neurodegenerative disorders (Erny et al., 2015).

Nicotinamide adenine dinucleotide (NAD^+^) is an essential metabolite involved in various critical cellular processes, including ATP production, DNA repair, mitochondrial biogenesis, cell survival, and death (Fang et al., 2017). NAD^+^ depletion has been linked to aging and age-related diseases (Covarrubias et al., 2021). In recent years, it has been revealed that boosting NAD^+^ levels may be a promising treatment to slow down aging and ameliorate neurodegeneration (Braidy et al., 2019). Nicotinamide riboside (NR) is a natural precursor for NAD^+^, which increases the intracellular level of NAD^+^ (Cantó et al., 2012). Our previous studies demonstrated that NR normalizes most key Alzheimer’s features, including mitophagy, neuroinflammation, DNA damage responses, and cognition decline in models of AD (Hou et al., 2018, 2021; Fang et al., 2019a). We found that NR treatment significantly increased the NAD^+^/NADH ratio in the cerebral cortex of both APP/PS1 mice and WT mice, improved the learning and memory ability in Morris water maze test and Y-maze test, and decreased neuroinflammation and cellular senescence in the 12-month-old AD brains (Hou et al., 2021). A recent study revealed a critical role for the gut microbiome in the metabolism of oral NR supplementation in mice (Shats et al., 2020). However, the role of NR supplementation on the gut microbiome in AD has not yet been studied. Using bacterial 16S ribosomal RNA (rRNA) gene sequencing of DNA isolated from fecal samples, we investigated the effects of 2 months of NR treatment on the gut microbiota of APP/PS1 transgenic mice. Here we found distinct changes in gut microbial diversity and taxonomical compositions in AD mice, and NR treatment normalized the altered diversity and richness of gut microbial communities.

## Materials and methods

### Animals

All mice were maintained on a standard NIH diet in a 12-h light/dark cycle in the National Institute on Aging (NIA), Baltimore. The rodent diet we used is T.2018SX.15 Global 18% Protein Extruded Rodent Diet (Sterilizable).^1^ The animals were group-housed if possible. All animal experiments were performed using protocols approved by the appropriate institutional animal care and use committee of the NIA. The APP/PS1 mice [B6:C3-Tg (Appswe, PSEN1dE9)85Dbo/Mmjax] were purchased from the Jackson Laboratory (Stock No. 004462) and the APP/PS1 heterozygous mice were maintained on C57BL/6J background. APP/PS1 (6 males and 3 females) and their littermates (WT) (9 males and 3 females) were used in this study. a widely used AD mouse model, and our previous studies and others have revealed that APP/PS1 mice develop Aβ pathology and behavioral deficits as early as 6 months old (Jankowsky et al., 2004; Ofengeim et al., 2017; Fang et al., 2019b; Gong et al., 2019) and are more significantly at 10–12 months old (Hou et al., 2021). Thus, 10 months old APP/PS1 mice and WT littermates were used in this study. APP/PS1 mice and WT mice (10-month-old) were given 12 mM NR (ChromaDex) in their drinking water *ad libitum* for 8 weeks, while the control groups received drinking water without NR as previously reported (Hou et al., 2018). Based on the estimated 7 ml water consumption per mouse per day and a 25 mg NR intake per mouse, we expect each mouse is getting a dose of 500 mg NR per kg per day. NR and water bottles were changed twice a week.

### Deoxyribonucleic acid extraction and sequencing

Fecal samples (one or two pellets) of each animal were collected individually in microfuge tubes on the same day and were quickly frozen until processed. Genomic DNA was extracted from the samples using the QIAamp PowerFecal Pro DNA Kit (QIAGEN, Hilden, Germany), following the manufacturer’s instructions. DNA extraction of the samples was analyzed by sequencing amplicons generated from the universal primers (341F and 806R) directed at the V3-V4 region of the 16S rRNA gene using the HiSeq Illumina platform. PCR products were purified using Agencourt AMPure XP beads (Beckman Coulter, Brea, CA, United States). The products were purified with Amplicons AMPure XP Beads (Beckman Coulter) according to the manufacturer protocol, and concentrations were estimated using Bioanalyzer and Qubitthe. Individually barcoded samples were mixed in equimolar amounts, and DNA sequencing adaptor indexes were ligated using the TruSeq DNA PCR-free LT Library Preparation Kit (Illumina, San Diego, CA, United States). Quality control was performed on an Agilent 2100 BioAnalyser using high sensitivity DNA chip. PhiX DNA (10%) was added to the denatured pools, and sequencing was performed on Illumina HiSeq 2500 System using the HiSeq V3 reagent kit. De-multiplexing and removal of indexes and primers were done with the Illumina software v. 2.6.2.1 on the instrument according to the standard Illumina protocol. Raw data were analyzed with the QIIME2 pipeline, version 2019.10. Contigs with 97% nucleotide sequence identity were clustered into operational taxonomic units (OTUs). The most abundant sequence in each OTU was chosen to represent its OTU. Taxonomy was assigned using the Greengenes database (v. 13_8) (DeSantis et al., 2006) and the RDP classifier (Wang et al., 2007).

### Statistical analysis of microbial communities

Statistical analyses were conducted on raw count data and data standardized to percent relative abundance. Alpha diversity was applied to analyze the complexity of the species diversity using R (V4.0.3) and the *vegan, dplyr, ggplot2* packages, including Good’s coverage, Observed OTUs, Chao1, Shannon diversity, inverse Simpson, ACE evenness and Pielou evenness (Thukral, 2017). To better visualize the differences among genotypes and treatments, Beta diversity metric was calculated by Principal Coordinate Analysis (PCoA) including the Bray-Curtis dissimilarity distance metrics (Minchin, 1987), using the *capscale* function implemented in the vegan R library *vegan* package, and by constraining for the variable of interest and conditioning for the remaining factors.^2^ The differences between groups were assessed by the permutational multivariate analysis of variance (PERMANOVA) (Anderson, 2001). The Venn plot was plotted using R package “*ggven*” (Gao et al., 2021). The enterotype analysis was performed with R packages *ade4*, *clusters*, and *vegan* (Dray and Dufour, 2007; see text footnote 2).^3^ The correlation network and LDA score were obtained from the MicrobiomeAnalyst website^4^ (Chong et al., 2020). Differential abundance of taxa among groups was determined at the OTU level in R using the *phyloseq* package (McMurdie and Holmes, 2013). Relative abundance comparisons at the genus, family, and phylum levels were performed on normalized OUT data. The other diagrams were visualized by the *ggplot2* package in R (V4.0.3).

### Statistical analysis

Data are expressed as boxplot with median, maximum, minimum, and the first and third quartiles. An unpaired two-tailed Student’s *t*-test (Welch two-sample *t*-test) was used to assess the differences between the two groups. Data sets involving more than two sets were evaluated using two-way ANOVA followed by Tukey’s multiple comparison tests. Correlations were analyzed using Spearman Correlation Coefficient. *P*-values were corrected for multiple testing with Benjamini–Hochberg false discovery rate correction (*q*-value). Data were analyzed using the R package (V4.0.3). The data plots as bar plots or dot plots were generated with the *ggplot2* package. Statistical significance was set at *P* < 0.05. Boxes represent the interquartile ranges (IQRs) between the first and third quartiles, and the line inside the box represents the median; whiskers represent the lowest or highest values within 1.5 times IQR from the first or third quartiles.

## Results

### Gut microbiome diversity decreased in Alzheimer’s disease mice compared with wild-type mice

**FIGURE 1:**
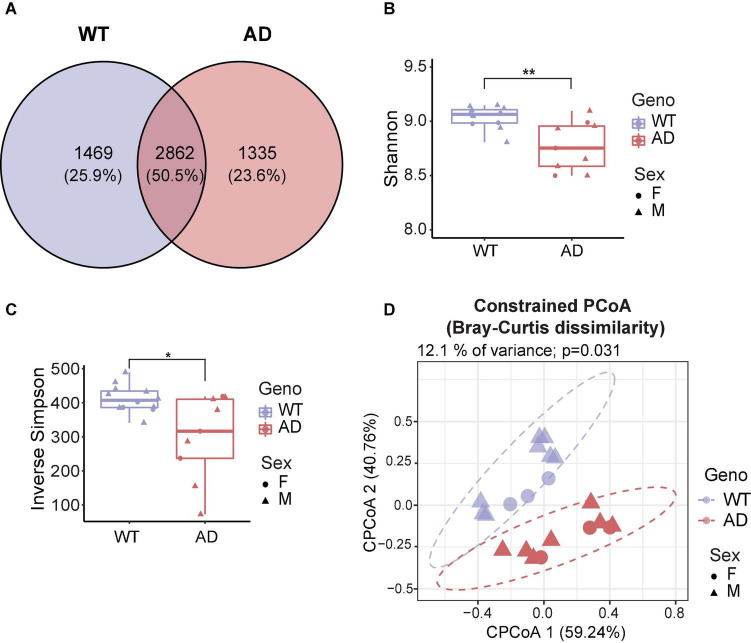
Gut microbiota diversity decreased in Alzheimer’s disease (AD) mice. **(A)** Venn diagram showed the unique and shared operation taxonomic units (OTUs) between AD and wild-type (WT) mice. **(B,C)** Alpha diversity is represented by Shannon diversity **(B)** and inverse Simpson **(C)** indices. *P < 0.05, **P < 0.01, Welch two-sample *t*-test. **(D)** Beta diversity is represented by constrained principal coordinate analysis (cPCoA) analysis using genotype as the constraining variable and ordinated with Bray–Curtis distances (12.1% of variance explained, *P* = 0.031). The percentage of variation indicated in each axis corresponds to the fraction of the total variance explained by the projection. Data are shown as all samples with a median value. F, females; M, males. *P < 0.05, **P < 0.01, AD (*n* = 9, 6 males and 3 females, 12-month-old) versus WT (*n* = 11, 9 males and 3 females, 12-month-old).

To investigate the differences in gut microbiota between 10-month-old APP/PS1 double transgenic mice (AD mice) and wild-type (WT) mice, we extracted the genomic DNA from the fecal samples and then sequenced it. Good’s coverage is a measure of the proportion of species and sequencing depth in a sample (Good, 1953). Our data showed that the Good’s coverage index of each group was over 99.995%, suggesting that vast majority of taxonomic units were detected in the samples (Supplementary Figure 1A). Bacterial operation taxonomic units (OTUs) were counted for each sample to express the richness of bacterial species with an identity cutoff of 97%. As a result, 2862 OTUs (50.5%) coexisted in both AD and WT mice, as demonstrated by the Venn diagram (Figure 1A). AD and WT mice have 23.6 and 25.9% unique OTUs, respectively, suggesting unique microbiome signatures between 2xTg AD and WT mice.

Microbial diversity provides overall information about changes in the composition of microorganisms. Alpha diversity reflects the richness and/or evenness of a sample (ter Braak, 1983). First, we investigated alpha diversity, assessed by observed OTUs, Chao1 index, Shannon diversity index, and inverse Simpson index (richness) and Pielou’s and ACE evenness (evenness). The number of unique taxa observed in each sample (observed OTUs) and the predicted number of rare organisms by the Chao1 index showed no difference between AD and WT mice (Supplementary Figures 1B,C). Also, the AD and WT mice had comparable Pielou’s and ACE evenness (Supplementary Figures 1D,E). Notably, significant differences in the Shannon diversity index and inverse Simpson index were observed based on measurements of both richness and evenness (Kim et al., 2017; Figures 1B,C), suggesting that AD mice had a decreased alpha diversity compared to WT mice.

Beta diversity is a measurement of the dissimilarity of two communities (ter Braak, 1983). To assess how affected the clustering of animals was by genotypes, the conditioned constrained principal coordinate analysis (cPCoA) was performed using Bray Curtis distance matrix (Anderson and Willis, 2003). As a result, we found a distinct dissimilarity between AD and WT mice (Figure 1D). This explained 12.1% of the total variance and revealed a prominent effect of the host genotype on bacterial communities. Together, these data demonstrate the significant differences in the gut microbiota diversity between AD and WT mice.

### Gut microbial composition changed in Alzheimer’s disease mice compared with wild-type mice

**FIGURE 2:**
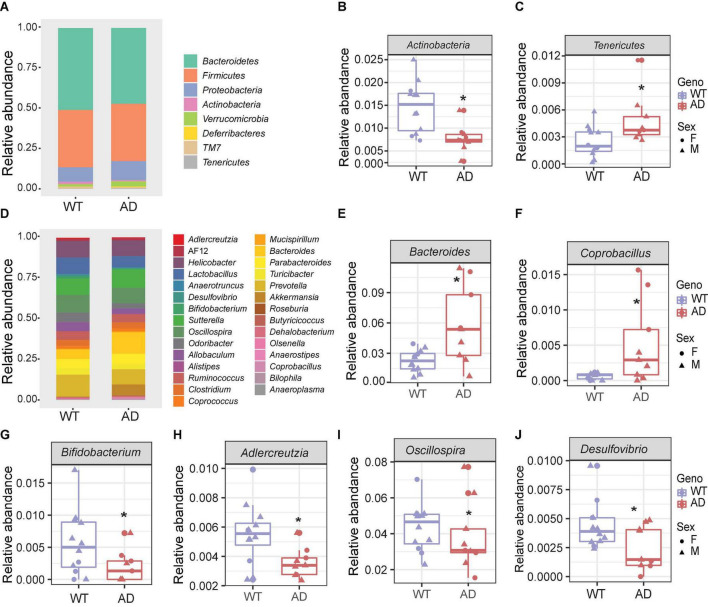
The gut microbial compositions of Alzheimer’s disease (AD) mice differed from wild-type (WT) mice. **(A)** Stacked bar chart showing the microbiome composition at the phylum level. **(B,C)** The relative abundances of *Actinobacteria* **(B)** decreased, and *Tenericutes* **(C)** increased in AD mice compared with WT mice. *P < 0.05, AD (*n* = 9) versus WT (*n* = 11); Welch two-sample *t*-test. **(D)** Stacked bar chart showing the microbiome composition at the genus level. **(E–J)** The relative abundances of genera *Bacteroides*, *Coprobacillus*, *Bifidobacterium*, *Adlercreutzia*, *Oscillospira*, and *Desulfovibrio*. *P < 0.05; Welch two-sample *t*-test. Data are shown as all samples with a boxplot. F, females; M, males; *P < 0.05, AD (*n* = 9, 6 males and 3 females, 12-month-old) versus WT (*n* = 11, 9 males and 3 females, 12-month-old).

The five major phyla, including *Bacteroidetes*, *Firmicutes*, *Proteobacteria*, *Actinobacteria*, and *Verrucomicrobia*, account for about 99% of the gut microbiota (Figure 2A). There was no significant difference in the two core phyla, *Firmicutes* and *Bacteroidetes*, between AD and WT mice. However, we found a lower abundance of *Actinobacteria* and a higher abundance of *Tenericutes* in AD mice than in WT mice, suggesting that these low-abundant taxa may be the major drivers of differences in the gut bacterial community composition (Figures 2B,C).

At the genus level, the gut microbial communities were more dramatically changed in AD mice (Figure 2D). The relative abundances of the genera *Bacteroides* and *Coprobacillus* were increased in AD mice, while *Bifidobacterium, Adlercreutzia, Oscillospira*, and *Desulfovibrio* were decreased in AD mice compared with WT mice (Figures 2E–J). Among these, *Bacteroides* were increased more than twofold in the AD intestines. *Coprobacillus*, which is associated with inflammation (Terzo et al., 2020), barely existed in WT mice, but were significantly increased in AD mice. *Bifidobacterium* and *Adlercreutzia*, which are known as probiotic microbes (Volokh et al., 2019), were significantly reduced in AD mice. Collectively, these altered gut microbial compositions may contribute to AD pathology.

### Enterotypes of the gut microbiome differed between Alzheimer’s disease and wild-type mice

**FIGURE 3:**
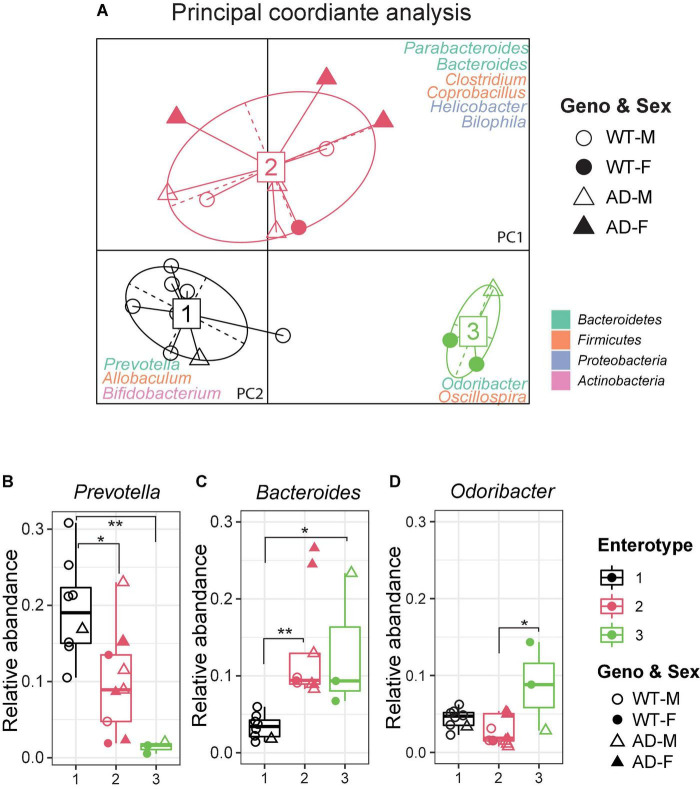
Enterotypes of the gut microbiome differed between Alzheimer’s disease (AD) and wild-type (WT) mice. **(A)** Enterotype analysis at the genus level of the gut microbiomes of AD mice (*n* = 9, 6 males and 3 females, 12-month-old) and WT mice (*n* = 11, 9 males and 3 females, 12-month-old). The data were most naturally separated into three clusters, as determined by the Calinski–Harabasz (CH) index and represented using principal coordinate analysis (PCoA). The corresponding phylum is labeled by colors separately. **(B–D)** Representative genera of each enterotype: Enterotype 1 is represented by *Prevotella*, Enterotype 2 is represented by *Bacteroides*, and Enterotype 3 is represented by *Odoribacter*. Data are shown as all samples with a boxplot. F, females; M, males. *P < 0.05, **P < 0.01, one-way analysis of variance (ANOVA) followed by Tukey’s multiple comparisons.

The gut microbiome can be clustered into distinct bacterial taxa according to an abundance of signature genera, known as enterotypes (Wang et al., 2014). In the human gut microbiota, there are three enterotypes: *Prevotella* (enterotype 1), *Bacteroides* (enterotype 2), and *Ruminococcus* (enterotype 3), all linked to long-term dietary patterns but independent of age, body mass index (BMI), or sex (Arumugam et al., 2011). Recently, the enterotypes were also observed in various animal models, including bees, pigs, chimpanzees, and mice (Moeller et al., 2012; Wang et al., 2014; Li et al., 2015; Xu et al., 2021). It is useful to describe the gut microbial community landscape using the enterotypes and it may be relevant in clinical practice. To identify the enterotypes at the genus level in AD and WT mice, a principal coordinate analysis (PCoA) was performed by Jensen–Shannon distance (JSD) (Figure 3A). Interestingly, most AD mice clustered in enterotype 2, which contained more *Bacteroides*. In contrast, most WT mice clustered in enterotype 1, which is dominated by *Prevotella*. The relative abundance of represented genera of each enterotype is shown in Figures 3B–D. Correlation network analysis also showed that genera in enterotype 2 (*Bacteroides*, *Clostridium*, *Coprobacillus*, and *Parabacteroides*) positively correlated with AD mice compared with WT mice (Supplementary Figure 1F), indicating that the gut microenvironment in AD mice was disturbed and differed significantly from WT mice.

### Sex difference exhibited in the gut microbiome of Alzheimer’s disease mice

**FIGURE 4:**
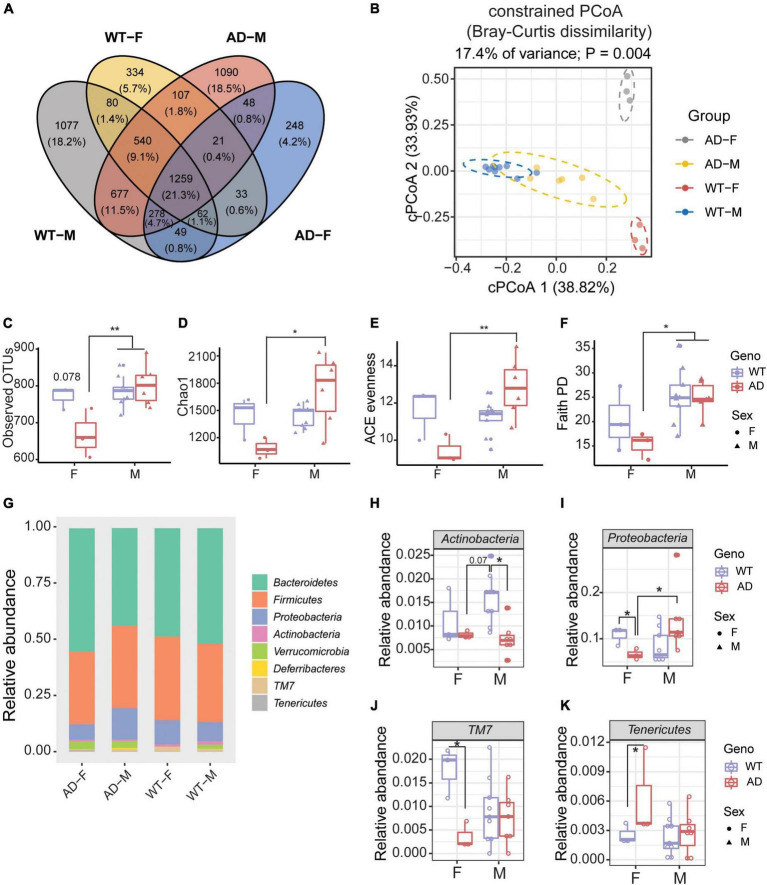
Sex differences exhibited in the gut microbiome of Alzheimer’s disease (AD) mice. **(A)** Venn diagram depicting the unique and shared operation taxonomic units (OTUs) between males and females of 2xTg AD and wild-type (WT) mice. **(B)** Beta diversity is represented by constrained principal coordinate analysis (cPCoA) analysis using genotype as the constraining variable and ordinated with Jaccard dissimilarity. **(C,D)** The richness of gut microbiome diversity is represented by observed OTUs **(C)** and Chao1 **(D)** indices (*n* = 3–6). **(E,F)** The evenness of gut microbiome diversity is represented by ACE evenness **(E)** and faith PD **(F)** (*n* = 3–6). *P < 0.05, **P < 0.01; two-way analysis of variance (ANOVA) followed by Tukey’s multiple comparisons. **(G)** Microbiome composition at the phylum level (*n* = 3–6). **(H–K)** The relative abundance of phyla of male and female mice in both 2xTg AD and WT groups (*n* = 3–6). *P < 0.05, **P < 0.01; two-way ANOVA followed by Tukey’s multiple comparisons. Data are shown as all samples with a boxplot. F, females; M, males. AD, 6 males and 3 females; WT, 9 males and 3 females; all mice were 12-month-old.

Next, we explored whether there was a sex difference in the gut microbiome between AD and WT mice. A Venn diagram showed that only 21.3% OTUs were shared between male and female WT and AD mice (Figure 4A). Males differed from females in their gut microbial structure, as measured by cPCoA analysis (Figure 4B, *p* = 0.004). For the alpha diversity, no difference was exhibited between female and male WT mice. However, the female AD mice exhibited lower alpha diversity indices compared with AD male mice (Observed OTUs, Chao1, ACE evenness, and faith phylogenetic distance) (Figures 4C–F). Moreover, we also found differences in the microbial compositions exhibited in the female AD mice compared with the other groups (Figure 4G). The relative abundance of *Actinobacteria* was lower in both female and male AD mice compared with male WT mice (Figure 4H). *Proteobacteria* were less abundant in female AD mice than in male AD mice (Figure 4I). Additionally, *Proteobacteria* and *TM7* decreased in female AD mice compared to female WT mice, while *Tenericutes* increased in female AD mice (Figures 4I–K). These results demonstrated a sex-specific difference in gut microbiota composition in AD mice.

### Nicotinamide riboside increased microbial diversity in Alzheimer’s disease mice after 1 and 8 weeks

It has been reported that the mitochondrial NADH/NAD^+^ ratio and ATP level remarkably diminish in the colonocytes of germ-free mice, suggesting an important role of the gut microbiome in energy homeostasis (Donohoe et al., 2011). Likewise, a recent study revealed that the gut microbiota was required for NAD^+^ metabolism with orally delivered NR supplementation (Shats et al., 2020). A significant NAD^+^ depletion was observed in the brains of AD mice in our previous studies (Fang et al., 2019b; Hou et al., 2021). Supplementation with the NAD^+^ precursor, NR, can efficiently increase brain NAD^+^ levels and alleviate brain features of AD (Hou et al., 2018, 2021). However, the effects of oral NR treatment on gut microorganisms in AD animal models are unknown.

**FIGURE 5:**
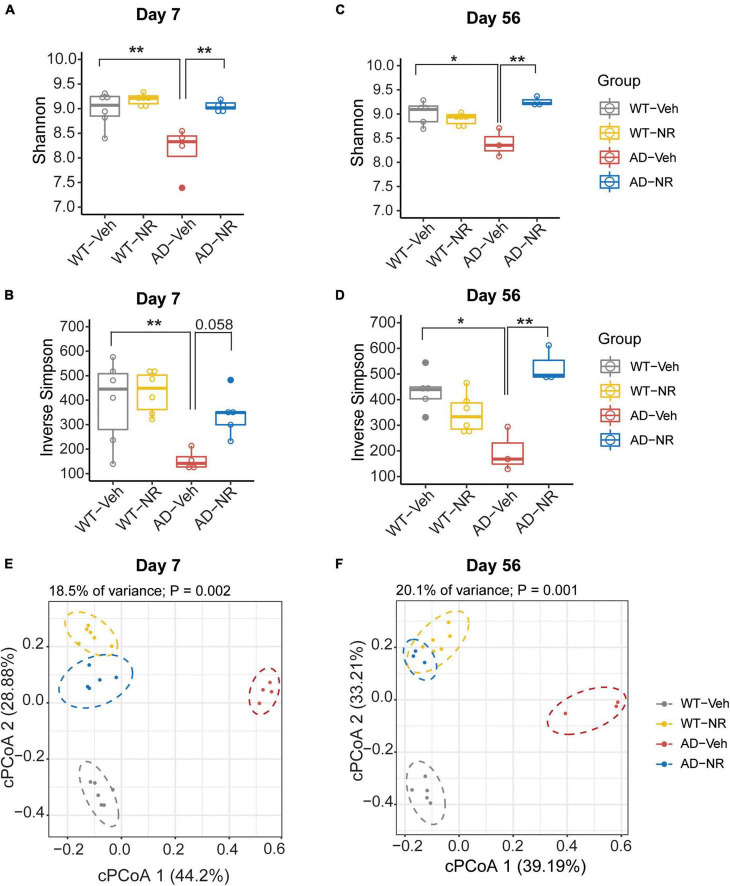
Microbial diversity changed after 1 and 8 weeks of nicotinamide riboside (NR) treatment. **(A–D)** Alpha diversity is represented by Shannon diversity and inverse Simpson on Day 7 and Day 56, respectively. *n* = 3–6, *P < 0.05, **P < 0.01, two-way analysis of variance (ANOVA) followed by Tukey’s *post-hoc* test. Data are shown as all samples with a boxplot. **(E,F)** Beta diversity is represented by constrained principal coordinate analysis (cPCoA) analysis using genotype and treatment as the constraining variables and ordinated with Jaccard dissimilarity on Day 7 **(E)** and Day 56 **(F)**, respectively. For Day 7, WT-Veh (5 males and 1 female), WT-NR (4 males and 2 females), AD-Veh (3 males and 1 female), AD-NR (3 males and 2 female), 10-month-old; For Day 56, WT-Veh (4 males and 1 female), WT-NR (4 males and 2 females), AD-Veh (2 males and 1 female), AD-NR (2 males and 1 female), 12-month-old.

To explore the effects of NR treatment on the gut microbiome, we treated mice with NR for 8 weeks and collected fecal samples of each mouse on Day 7 and Day 56. There were no significant differences between WT-Veh, WT-NR, and AD-NR. However, we observed a significant reduction of alpha diversity (Shannon diversity index and inverse Simpson index) on Day 7 and Day 56 in AD mice, which was reversed by NR treatment (Figures 5A–D). Again, the microbiota composition was significantly different between AD-vehicle (AD-Veh) and WT-vehicle (WT-Veh) mice in cPCoA analysis with distance calculated by Bray-Curtis dissimilarity (Figures 5E,F). AD-Veh mice are clustered separately from WT-Veh mice. Surprisingly, the two clusters of AD and WT mice with NR treatment were much closer to each other (AD-NR and WT-NR in Figures 5E,F), suggesting that NR treatment made the gut microbiota more similar regardless of genotype.

**FIGURE 6:**
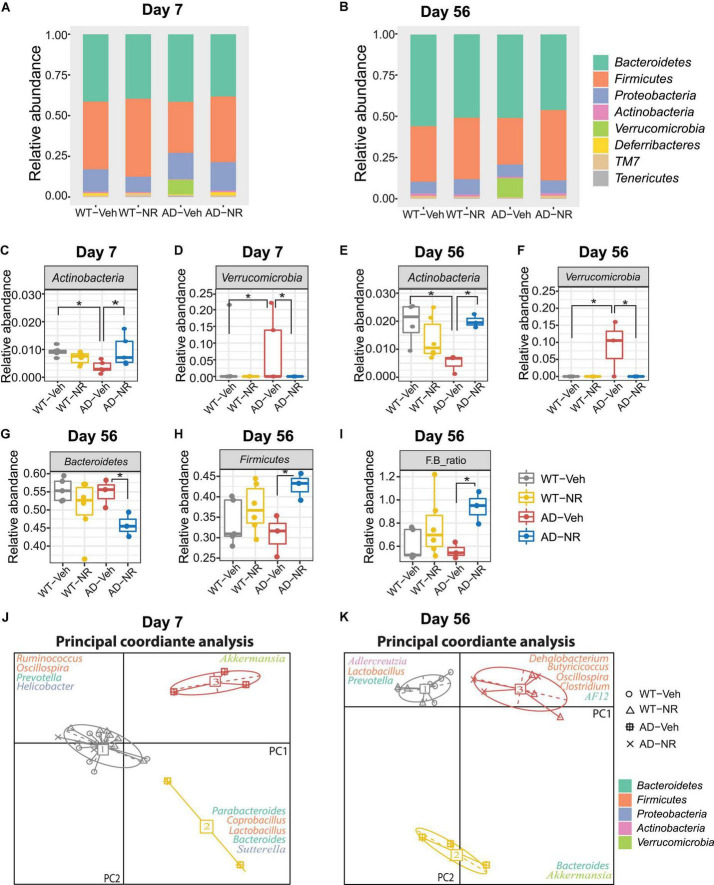
Microbial communities changed after 1 and 8 weeks of nicotinamide riboside (NR) treatment. **(A,B)** Microbiome composition at the phylum level on Day 7 **(A)** and Day 56 **(B)**. **(C–F)** The changes in relative abundances of *Actinobacteria* and *Verrucomicriobia* on Day 7 **(C,D)** and Day 56 **(E,F)** (*n* = 3–6). Data showed as mean ± SD. *P < 0.05, two-way analysis of variance (ANOVA) followed by Tukey’s multiple comparisons. **(G–I)** The changes in relative abundances of *Bacteroidetes* **(G)**, *Firmicutes* **(H)**, *and* the F/B ratio **(I)** on Day 56 (*n* = 3–6). Data showed as mean ± SD. *P < 0.05, two-way ANOVA followed by Tukey’s multiple comparisons. **(J,K)** Enterotype analysis at the genus level of the gut microbiomes on Day 7 **(J)** and Day 56 **(K)**, respectively. The data are most naturally separated into three clusters, as determined by the CH index and represented using principal coordinate analysis (PCoA) (*n* = 3–6). Data are shown as all samples with a median value. *P < 0.05. For Day 7, WT-Veh (5 males and 1 female), WT-NR (4 males and 2 females), AD-Veh (3 males and 1 female), AD-NR (3 males and 2 female), 10-month-old; For Day 56, WT-Veh (4 males and 1 female), WT-NR (4 males and 2 females), AD-Veh (2 males and 1 female), AD-NR (2 males and 1 female), 12-month-old.

The relative abundance changes at the phylum level on Day 7 and Day 56 are separately shown in Figures 6A,B. Interestingly, NR treatment reversed the previously observed decreased abundance in *Actinobacteria* in AD-Veh mice on Day 7 and Day 56, while there were no differences between WT-Veh, WT-NR, and AD-NR (Figures 6C,E). Moreover, the higher content of *Verrucomicrobia* was also reversed on Day 7 and Day 56 after NR treatment (Figures 6D,F).

It was reported that LPS, as a component from *Bacteroidetes* in the host, could induce cognitive dysfunction and inflammation in AD brains (Zhao et al., 2017). Similar results were displayed in mild cognitively impaired patients, who with more *Bacteroidetes* had lower logical memory subtest scores, indicating that higher *Bacteroidetes* is related to cognitive impairment (Saji et al., 2019). However, we did not observe this trend of *Bacteroidetes* in AD mice compared with WT mice. Instead, we found that *Bacteroidetes* decreased after NR treatment only in AD mice while having no effects on WT mice on Day 56 (Figure 6G). The reduced *Bacteroidetes* in the AD mice after NR administration may be related to the improvement of memory observed in our previous studies (Hou et al., 2018, 2021). We also found that NR increased *Firmicutes* in AD mice after 8 weeks of treatment (Figure 6H). Lower abundances of *Actinobacteria* and *Firmicutes* were found in humans with inflammatory bowel disease (Rehman et al., 2010; Willing et al., 2010). Therefore, we speculate that these two phyla observed in AD mice may be linked to inflammation. In addition, a low *Firmicutes* to *Bacteroidetes* (F/B) ratio is believed to be correlated with inflammation (Rowin et al., 2017). In our data, the F/B ratio was elevated in AD mice after NR treatment (Figure 6I), indicating the potential anti-inflammatory effects of NR supplementation on AD mice.

Enterotype analysis suggested that AD-veh mice exhibited a distinct type from other groups on Day 7, as AD-veh mice are more likely to have the *Bacteroides*-dominated enterotype (Figure 6J enterotype 2). In contrast, the other three groups (WT-veh, WT-NR, AD-NR) clustered as the *Prevotella*-dominated enterotype (Figure 6J, enterotype 1). The enterotypes, dominated by *Prevotella* or *Bacteroides*, are predictive responses to diets and drugs (Christensen et al., 2018). It is believed that hosts with *Bacteroides*-dominated enterotype are more vulnerable to perturbations of the gut microbiome, especially coming from diets or drugs (Vieira-Silva et al., 2016). Subjects designated as *Prevotella* enterotype were more likely to consume a high fiber diet, and the main products of bacterial fermentation of *Prevotella* enterotype are short chain fatty acids (SCFAs), which can serve as metabolites that regulate gut-brain communication (Silva et al., 2020). The different traits of the enterotypes may help us understand specific gut microbiome-dependent properties of AD. In line with this, we found that AD mice without NR treatment were still dominated by *Bacteroides*- and *Akkermansia*-dominated microbiota on Day 56, while WT mice without NR treatment were dominated by *Prevotella, Lactobacillus*, and *Adlercreutzia* (Figure 6K). Notably, AD and WT mice treated with NR supplementation clustered together, significantly separating from groups without NR treatment (Figure 6K, enterotype 2). The representative genera were *Oscillospira, Clostridium, Dehalobacteriumm, Butyricicoccus*, and *AF12*, which are known as commensal probiotics and contribute to anti-inflammatory benefits. Here, we found that NR treatment boosted these probiotics in AD mice, suggesting NR treatment possibly made the intestinal environment more healthy and less inflammatory.

### Altered genera are correlated with nicotinamide riboside treatment in Alzheimer’s disease mice

**FIGURE 7:**
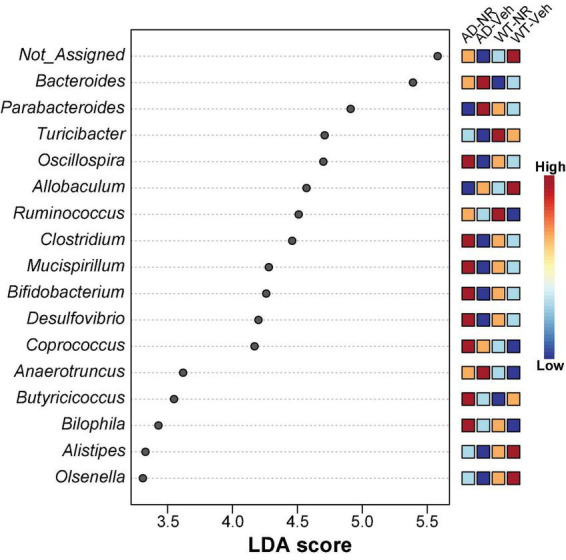
Potential microbiome biomarker of NR treatment on Alzheimer’s disease (AD) mice at the genus level. The Linear discriminant analysis (LDA) effect size (Lefse) scores were computed for features differentially abundant between groups. Here we showed the top 15 most significant biomarkers (*P* < 0.001). The right heatmap shows the relative abundance (log_10_ transformation) of operation taxonomic units (OTUs). The left histogram shows the featured microbiome biomarker at the genus level. WT-Veh (4 males and 1 female), WT-NR (4 males and 2 females), AD-Veh (2 males and 1 female), AD-NR (2 males and 1 female), 12-month-old.

We applied Linear discriminant analysis Effect Size (LEfSE) analysis to identify specific bacterial taxa changes induced by 8 weeks of NR treatment. As shown in Figure 7, the LDA scores of *Bacteroides*, *Parabacteroides*, and *Anaerotruncus* were higher in AD mice, while *Turicibacter*, *Oscillospira*, *Clostridium*, *Mucispirillum*, *Bifidobacterium*, *Desulfovibrio, Alistipes*, and *Olsenella* were lower in AD mice, compared with controls. Next, we compared genus-level differences after 8 weeks of NR treatment. The relative abundance of species at the phylum, family, genus, and species level in these groups after 8 weeks NR treatment were shown in Tables 1–4. The genera *Oscillospira* (family *Ruminococcaceae*), *Butyricicoccus* (family *Ruminococcaceae*), *Desulfovibrio* (family *Desulfovibrionaceae*), *Bifidobacterium* (family *Bifidobacteriaceae*), *Olsenella* (family *Coriobacteriaceae*), and *Adlercreutzia* (family *Coriobacteriaceae*) were less abundant in the AD vehicle group, and NR increased their abundances after 8 weeks of treatment (Supplementary Figures 2A–F). The elevated genera *Bacteroides* (family *Bacteroidaceae*), *Akkermansia* (family *Verrucomicrobiaceae*), and *Lactobacillus* (family *Lactobacillaceae*) observed in AD mice were reversed after NR treatment (Supplementary Figures 2G–I).

**TABLE 1:** The relative abundance (%) analysis at phylum level.

Phylum	WT-Veh	WT-NR	AD-Veh	AD-NR
*p__Actinobacteria*	1.95 ± 0.75*	1.37 ± 0.75	0.51 ± 0.34	1.99 ± 0.24*
*p__Bacteroidetes*	56.29 ± 3.06	50.58 ± 7.8	55.62 ± 2.59	45.52 ± 3.48*
*p__Firmicutes*	32.35 ± 5.36	37.27 ± 5.83	28.25 ± 3.18	42.71 ± 3.31*
*p__Proteobacteria*	7.5 ± 4.09	9.33 ± 4.32	7.36 ± 1.76	7.69 ± 0.55
*p__Tenericutes*	0.11 ± 0.07	0.42 ± 0.26	0.23 ± 0.22	0.29 ± 0.21
*p__Deferribacteres*	0.11 ± 0.08	0.19 ± 0.12	0.02 ± 0.03	0.26 ± 0.11
*p__Verrucomicrobia*	0 ± 0*	0 ± 0*	8.84 ± 8.13	0 ± 0*
*p__TM7*	1.22 ± 1	0.65 ± 0.44	0.45 ± 0.48	1.2 ± 0.48
*p__Cyanobacteria*	0.06 ± 0.12	0.06 ± 0.08	0.13 ± 0.22	0.06 ± 0.03

Values are expressed as mean ± SD. *P*-values were corrected for multiple testing with Benjamini–Hochberg false discovery rate correction (*q*-value). *P < 0.05, versus AD-Veh group. WT-Veh (4 males and 1 female), WT-NR (4 males and 2 females), AD-Veh (2 males and 1 female), AD-NR (2 males and 1 female), 12-month-old.

**TABLE 2:** The relative abundance (%) of species at family level.

Family	WT-Veh	WT-NR	AD-Veh	AD-NR
*f__Coriobacteriaceae*	0.82 ± 0.38*	0.87 ± 0.35	0.37 ± 0.29	1.39 ± 0.36**
*f__Rikenellaceae*	2.99 ± 1.7	2.79 ± 1.29	2.72 ± 1.55	2.71 ± 0.33
*f__Lachnospiraceae*	9.14 ± 2.98	12.17 ± 4.9	9.32 ± 1.01	14.05 ± 2.68*
*f__Helicobacteraceae*	1.84 ± 1.14	4.75 ± 6.07	2.52 ± 0.99	2.81 ± 0.71
*f__S24.7*	45.18 ± 2.51**	38.8 ± 7.74	35.28 ± 8.1	39.91 ± 3.22*
*f__Ruminococcaceae*	7.19 ± 1.31*	9.9 ± 3.55	5.38 ± 1.5	11.57 ± 1.08**
*f__Lactobacillaceae*	4.56 ± 1.66	2.52 ± 1.82	4.86 ± 4.96	2.75 ± 0.88
*f__Desulfovibrionaceae*	0.88 ± 0.14*	0.81 ± 0.59	0.37 ± 0.21	1.21 ± 0.44*
*f__Bifidobacteriaceae*	1.04 ± 0.8	0.62 ± 0.67	0.47 ± 0.36	0.58 ± 0.82
*f__Mogibacteriaceae*	0.26 ± 0.07	0.29 ± 0.14	0.28 ± 0.09	0.47 ± 0.32
*f__Alcaligenaceae*	5.32 ± 4.35	5.02 ± 1.7	4.63 ± 1.94	5.02 ± 0.43
*f__Odoribacteraceae*	2.76 ± 2.51	2.11 ± 0.89	1.47 ± 0.32	5.04 ± 2.1
*f__Erysipelotrichaceae*	2.54 ± 1.73	2.38 ± 1.44	2.6 ± 0.78	2.32 ± 1.56
*f__Clostridiaceae*	0.74 ± 0.97	0.67 ± 1.17	0.01 ± 0.02	0.65 ± 0.95
*f__Deferribacteraceae*	0.31 ± 0.42	0.22 ± 0.14	0.09 ± 0.13	0.32 ± 0.15*
*f__Bacteroidaceae*	2.53 ± 1.11*	3.35 ± 3.22	4.33 ± 3.05	2.89 ± 1.82*
*f__Porphyromonadaceae*	1.96 ± 0.74	2.36 ± 1.07	2.6 ± 2.5	1.44 ± 0.11
*f__Turicibacteraceae*	0.76 ± 0.51	1.12 ± 1.79	0.14 ± 0.28	0.73 ± 1
*f__Prevotellaceae*	0.54 ± 0.4*	0.71 ± 0.65	1.18 ± 0.4	0.41 ± 0.25*
*f__Paraprevotellaceae*	6.77 ± 3.35*	6.68 ± 7.45	9.81 ± 6.69	4.33 ± 4.29*
*f__Verrucomicrobiaceae*	0.01 ± 0.03*	0 ± 0	9.87 ± 11.46	0 ± 0**
*f__F16*	1.35 ± 1.06	0.75 ± 0.51	0.79 ± 0.42	1.45 ± 0.63
*f__Mycoplasmataceae*	0.05 ± 0.08	0.27 ± 0.16	0.21 ± 0.22	0.1 ± 0.11
*f__Peptostreptococcaceae*	0.31 ± 0.41	0.24 ± 0.39	0.06 ± 0.11	0.18 ± 0.31
*f__Dehalobacteriaceae*	0.33 ± 0.11*	0.39 ± 0.11	0.24 ± 0.17	0.49 ± 0.12*
*f__Peptococcaceae*	0.1 ± 0.06*	0.11 ± 0.08	0.01 ± 0.03	0.15 ± 0.09*
*f__Anaeroplasmataceae*	0 ± 0	0.08 ± 0.11	0 ± 0	0.05 ± 0.08**

Values are expressed as mean ± SD. *P*-values were corrected for multiple testing with Benjamini–Hochberg false discovery rate correction (*q*-value). *P < 0.05, **P < 0.01, versus AD-Veh group. WT-Veh (4 males and 1 female), WT-NR (4 males and 2 females), AD-Veh (2 males and 1 female), AD-NR (2 males and 1 female), 12-month-old.

**TABLE 3:** The relative abundance (%) analysis at the genus level.

Genus	WT-Veh	WT-NR	AD-Veh	AD-NR
*g__Adlercreutzia*	1.23 ± 0.43**	1.01 ± 0.67**	0.03 ± 0.02	1.74 ± 0.47**
*g__AF12*	1.24 ± 0.51	1.71 ± 1.05	0.93 ± 0.34	1.69 ± 0.51*
*g__Helicobacter*	4.36 ± 2.67	9.25 ± 10.31	4.88 ± 2.08	5.99 ± 1.26
*g__Lactobacillus*	10.73 ± 3.82*	5.52 ± 3.91	9.55 ± 9.46	5.84 ± 1.71
*g__Anaerotruncus*	0.11 ± 0.08*	0.41 ± 0.24#	0.28 ± 0.27	0.51 ± 0.13
*g__Desulfovibrio*	1.03 ± 0.1*	1.23 ± 0.61	0.51 ± 0.42	1.89 ± 1.04*
*g__Bifidobacterium*	2.9 ± 1.78*	1.36 ± 1.45	0.95 ± 0.77	3.25 ± 1.76**
*g__Sutterella*	12.4 ± 10.18	11.09 ± 4.22	8.89 ± 3.51	10.8 ± 1.38
*g__Oscillospira*	11.55 ± 4.59*	12.83 ± 4.51*	6.5 ± 2.21	16.07 ± 2.86*
*g__Odoribacter*	6.71 ± 6.53*	4.69 ± 2.34	10.72 ± 4.03	2.83 ± 0.76*
*g__Allobaculum*	5.52 ± 4.03	4.48 ± 3.22	3.61 ± 2.85	3.67 ± 3.23
*g__Alistipes*	0.32 ± 0.18	0.25 ± 0.11	0.25 ± 0.1	0.24 ± 0.07
*g__Ruminococcus*	4.94 ± 1.52	6.08 ± 1.09*	3.76 ± 1.47	7.58 ± 0.91*
*g__Clostridium*	4.5 ± 2.14*	5.06 ± 4.81	1.85 ± 1.02	4.81 ± 4.41
*g__Coprococcus*	1.37 ± 0.58	1.85 ± 0.57	0.84 ± 0.41	2.77 ± 0.62**
*g__Mucispirillum*	0.72 ± 0.97	0.46 ± 0.28	0.18 ± 0.31	0.67 ± 0.28*
*g__Bacteroides*	5.9 ± 2.33*	7.29 ± 7.34	17.96 ± 5.39	6.16 ± 3.72*
*g__Parabacteroides*	4.56 ± 1.66	4.93 ± 1.83	4.68 ± 4.41	3.09 ± 0.36
*g__Turicibacter*	1.79 ± 1.13*	2.77 ± 4.66	0.32 ± 0.64	1.64 ± 2.24
*g__Prevotella*	17.07 ± 8.06	15.49 ± 16.53	10.02 ± 9.24	15 ± 5.54
*g__Akkermansia*	0.03 ± 0.07*	0 ± 0*	28.05 ± 21.45	0 ± 0*
*g__Roseburia*	0.02 ± 0.04	0 ± 0	0 ± 0	0.19 ± 0.33
*g__Butyricicoccus*	0.22 ± 0.15*	0.33 ± 0.23	0.07 ± 0.13	0.44 ± 0.28*
*g__Dehalobacterium*	0.78 ± 0.27	0.86 ± 0.32	0.47 ± 0.33	1.07 ± 0.3*
*g__Olsenella*	0.25 ± 0.18*	0.31 ± 0.19	0.15 ± 0.12	0.49 ± 0.26*
*g__Anaerostipes*	0.01 ± 0.02	0 ± 0	0.03 ± 0.06	0 ± 0
*g__Coprobacillus*	0.12 ± 0.14*	0.41 ± 0.58	0.35 ± 0.11	0.22 ± 0.23
*g__Bilophila*	0.12 ± 0.19	0.16 ± 0.19	0.04 ± 0.05	0.36 ± 0.19*
*g__Anaeroplasma*	0 ± 0	0.17 ± 0.23	0 ± 0	0.1 ± 0.17

Values are expressed as mean ± SD. *P*-values were corrected for multiple testing with Benjamini–Hochberg false discovery rate correction (*q*-value). *P < 0.05, **P < 0.01, versus AD-Veh group. #*P* < 0.05, versus WT-Veh group. WT-Veh (4 males and 1 female), WT-NR (4 males and 2 females), AD-Veh (2 males and 1 female), AD-NR (2 males and 1 female), 12-month-old.

**TABLE 4:** The relative abundance (%) analysis at the species level.

Species	WT-Veh	WT-NR	AD-Veh	AD-NR
*s__C21_c20*	0.35 ± 0.08	0.53 ± 0.3##	0.31 ± 0.3	1.56 ± 0.38
*s__Pseudolongum*	1.28 ± 0.69*	0.49 ± 0.52	0.05 ± 0.08	0.98 ± 0.35*
*s__Guilliermondii*	0.04 ± 0.04	0.17 ± 0.17	0 ± 0	0.27 ± 0.3
*s__Schaedleri*	0.26 ± 0.35	0.19 ± 0.12	0.02 ± 0.03	0.27 ± 0.1
*s__Cocleatum*	0.06 ± 0.08	0.07 ± 0.1	0.12 ± 0.12	0.02 ± 0.04
*s__Gnavus*	0.75 ± 0.15	0.82 ± 0.49	0.63 ± 0.45	1.04 ± 0.24
*s__Methylpentosum*	0.15 ± 0.04*	0.14 ± 0.04*	0.06 ± 0.05	0.2 ± 0.02*
*s__Muciniphila*	0.01 ± 0.02**	0 ± 0**	11.65 ± 10.2	0 ± 0**
*s__Vaginalis*	0.47 ± 0.22	0.41 ± 0.33	0.59 ± 0.83	0.35 ± 0.12
*s__Animalis*	0.28 ± 0.46	0.04 ± 0.06	0.22 ± 0.28	0.05 ± 0.09
*s__Pullicaecorum*	0.08 ± 0.06	0.14 ± 0.11	0.04 ± 0.07	0.19 ± 0.11
*s__Hepaticus*	0.13 ± 0.07*	0.39 ± 0.21	0.45 ± 0.13	0.34 ± 0.13*
*s__Acidifaciens*	0.52 ± 0.2**	0.66 ± 0.62	2.2 ± 0.64	0.4 ± 0.13*
*s__Distasonis*	0.34 ± 0.16*	0.74 ± 0.32	1.55 ± 1.4	0.45 ± 0.14*
*s__Indistinctus*	0.12 ± 0.07	0.09 ± 0.04	0.13 ± 0.04	0.09 ± 0.03

Values are expressed as mean ± SD. *P*-values were corrected for multiple testing with Benjamini–Hochberg false discovery rate correction (*q*-value). *P < 0.05, **P < 0.01, versus AD-Veh group. ##*P* < 0.01, versus WT-Veh group. WT-Veh (4 males and 1 female), WT-NR (4 males and 2 females), AD-Veh (2 males and 1 female), AD-NR (2 males and 1 female), 12-month-old.

Furthermore, NR specifically increased the genera *Coprococcus* (family *Lachnospiraceae*), *Ruminococcus* (family *Lachnospiraceae*), *Odoribacter* (family *Odoribacteraceae*), *Mucispirillum* (family *Deferribacteraceae*), *AF 12* (family *Rikenellaceae*), and *Bilophila* in AD mice, but not in WT mice (Supplementary Figures 2J–O).

Overall, these altered bacterial communities indicate a fragile gut microbiome composition in AD mice. Interestingly, most of the changed gut microbiome in AD gut flora compared to WT were reversed after NR treatment. Added to our previous observations on anti-neuroinflammation and cognition improvement, it is suggested that NAD^+^ supplementation has profound benefits on the gut microbiome in AD mice.

## Discussion

The complex gut microbiome undergoes rapid changes with lifestyle, diet, medication use, exercise, sleep, and stress (Conlon and Bird, 2015; Priya and Blekhman, 2019). Accumulating research studies suggest that disruption of the bidirectional communication between intestinal microbiota and the brain may contribute to the pathogenesis of various neurological and mental diseases, including AD. Loss of diversity in core microbiota groups is associated with increased gut permeability, frailty, inflammation, and reduced cognitive performance (O’Toole and Jeffery, 2015). Importantly dietary interventions, which modulate the gut and brain are now beginning to be elucidated. A recent study has shown that NR alleviates alcohol-induced depressive-like behaviors in mice by altering the gut microbiota composition (Jiang et al., 2020). Our previous findings have demonstrated that NR supplementation maintains mitochondrial function, counters oxidative stress and neuroinflammation, and improves learning and memory in AD animal models (Hou et al., 2018, 2021; Fang et al., 2019a). Another study on oral NR supplementation in elderly adults also revealed an anti-inflammatory effect of NR (Elhassan et al., 2019). Given that oral administration is the main form of NR treatment, it is important to study the potential bidirectional influences of NR on the gut microbiome in AD.

In this study, we identified that the gut of AD mice exhibited decreased microbial diversity and a distinct composition compared with WT mice. Interestingly, female AD mice had lower microbial diversity than male AD mice, suggesting sex differences is a risk factor in AD-related gut microbiome changes. In line with this finding, females are more impacted by AD than males in both human and animal models (Sohn et al., 2018; Alzheimer’s-Association, 2021). The sex-specific pattern of gut microbial changes observed in female AD mice may be one of the causes of this disease heterogeneity.

Rather than simply counting the number of bacterial species in the gut, a more comprehensive analysis of enriched and depleted microbial taxa and diversity alterations was employed to define the disease state. We observed significantly decreased *Actinobacteria* in AD mice at the phylum level. Although *Actinobacteria* contribute to a small fraction of the total gut bacteria, it plays a pivotal role in the development and maintenance of gut homeostasis, including the maintenance of the intestinal barrier, production of SCFA, modulating inflammatory responses, and the gut-brain axis (Binda et al., 2018). *Actinobacteria* are more abundant in young human adults and show a decreasing trend with age (Li et al., 2021). Similar changes have been observed in aged mice, where *Actinobacteria* decreased and *Verrucomicrobia* increased (Zhang et al., 2017). *Actinobacteria* also negatively correlate with inflammatory-related depression, suggesting a role for *Actinobacteria* in normal mental health (Huang et al., 2019). Taken together, these studies indicate that a relatively high level of *Actinobacteria* is associated with health conditions in humans and mice, and decreased levels are associated with aging and diseases. Consistent with this, the decreased abundance of *Actinobacteria* observed in AD mice in this study suggests that *Actinobacteria* may be associated with AD pathology, perhaps through its role in inflammation. Notably, *Bifidobacterium*, belonging to the *Actinobacteria* phylum in the human gut, is widely used as a probiotic (Ramakrishna, 2013). Decreased *Bifidobacterium* was reported in the microbiome of AD patients (Vogt et al., 2017). It was also reported that oral administration of the probiotic *Bifidobacterium* species could counteract inflammation (Veiga et al., 2010). In our previous studies, we found increased inflammation in the brain of AD mice, and NR treatment could decrease the inflammation (Hou et al., 2021). Accordingly, we found that *Bifidobacterium* was decreased in AD mice (Figure 2), and NR treatment efficiently increased its abundance (Supplementary Figure 2D). For the phylum *Tenericutes*, a previous study also found that the 8 months old APP/PS1 mice had more *Tenericutes* compared to controls (Harach et al., 2017), which is in accord with our finding (Figure 2C). Our previous study had shown impaired learning and memory and decreased synaptic plasticity in the AD mice (Hou et al., 2021). Interestingly, increased phylum *Tenericutes* was found to be associated with cognitive impairment (Yang et al., 2020). Our microbial data further confirmed that increased *Tenericutes* might contribute to impaired memory ability in AD mice.

*Firmicutes* and *Bacteroidetes* are two dominant phyla representing ∼90% of the total gut microbiota (Thursby and Juge, 2017). The F/B ratio is suggested to be a critical index reflecting intestinal health (Stojanov et al., 2020). The *Firmicutes* contain many SCFA- and butyrate-producing species, which regulate the immune system and gut barrier integrity (Vaiserman et al., 2020). Moreover, increased *Firmicutes* are associated with obesity and an increased capacity for dietary energy harvest (Turnbaugh et al., 2006). However, controversy about the F/B ratio theory remains, and many studies have failed to find consistent results (Schwiertz et al., 2010; Escobar et al., 2014; Yasir et al., 2015; Gao et al., 2018). In a previous study, a significant reduction in *Firmicutes* and an increase in *Bacteroidetes* were found in 8-month-old APP/PS1 mice (Harach et al., 2017), while the opposite trend was displayed in the 24-month-old APP/PS1 mice in another study (Bauerl et al., 2018). However, in our study, we did not find significant differences in *Firmicutes* or *Bacteroidetes* between the 12-month-old AD and WT mice. The inconsistent results observed in our studies and others may be ascribed to the background, sex, or age differences in the mice, and the specific link of *Firmicutes* and *Bacteroidetes* with different ages and genders in APP/PS1 mice remains unknown and requires more extensive investigations. In AD patients, gut microbial alterations with decreased *Firmicutes* and increased *Bacteroidetes* were observed (Vogt et al., 2017). While abundances of *Firmicutes* and *Bacteroidetes* were similar between the AD-Veh and WT-Veh mice, we did find increased *Firmicutes* and decreased *Bacteroidetes* in AD mice after NR treatment (Figures 6G,H). Considering the essential roles of *Firmicutes* and *Bacteroidetes* in energy homeostasis (Rosenbaum et al., 2015), we speculate that the oral administration of NR may have a specific impact on the energy metabolism of host-microbiota exclusively in AD mice. However, future studies are needed to further explain the reasons behind these differing findings.

**FIGURE 8:**
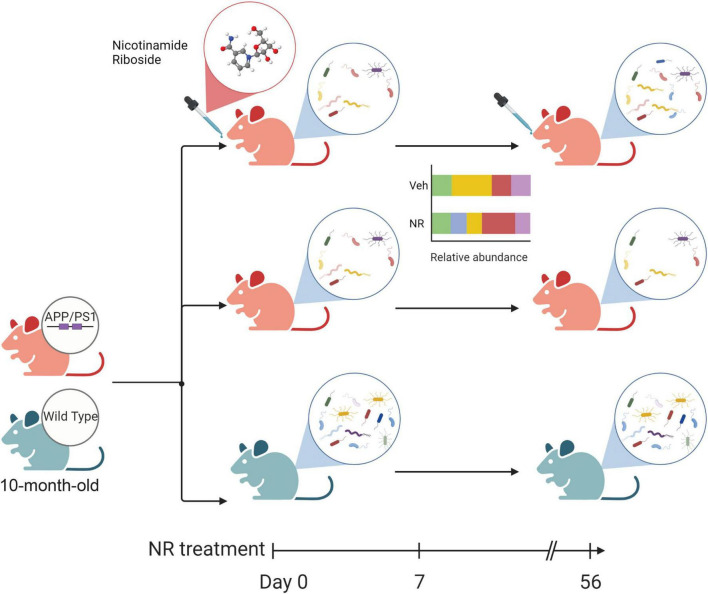
The schematic diagram of the main changes in the gut microbiome associated with nicotinamide riboside (NR) treatment in Alzheimer’s disease (AD) mice. The green mouse denotes the wild-type (WT) group, and the red mouse denotes the AD group in this study. Firstly, AD mice show decreased diversity compared with WT. NR could restore the richness and diversity of the gut microbiome in AD mice after 8 weeks of NR treatment. Additionally, NR could shift the relative abundance of some gut microbiomes which may favor an anti-inflammatory gut micro-environment, as shown in the simplified bar plot. NR can also specifically affect the gut flora of AD mice, as shown in Supplementary Figure 2. These changes may directly or indirectly influence the balance of intestinal flora and contribute to AD.

The alteration of microbial communities in disease and treatments provides insight into the symbiotic niche dynamics. In this study, we explored the effects of NR treatment on the gut microbiome for 8 weeks. A schematic diagram of the main gut microbiome changes associated with NR treatment in AD mice (Figure 8), shows that NR restores the decreased richness and diversity in AD mice. We also found several bacterial markers of gut dysbiosis in AD mice, and NR treatment had a significant beneficial effect on them. For example, one of the genera biomarkers is the *Bacteroides* (phylum *Bacteroidetes*). Previous studies have shown that elderly adults have a unique change in gut microbiome pattern compared with younger subjects, a high *Bacteroides* dominant microbial community, and a lower gut microbiome uniqueness (Wilmanski et al., 2021). Moreover, there is a positive correlation between the abundance of *Bacteroides* and all-cause mortality risk, implicating that *Bacteroides* might be a potential marker reflecting the health status of individuals (Wilmanski et al., 2021). In agreement with this, we found that the relative abundance of *Bacteroides* increased in AD mice and that NR treatment restored it back to normal levels, which may be associated with improved learning and memory in AD mice after NR treatment observed in our previous studies (Hou et al., 2018, 2021).

A subset of intestinal bacteria that ferment complex carbohydrate fibers to produce SCFAs and butyrate, such as *Bifidobacterium* (family *Bifidobacteria*), *Butyricicoccus*, and *Coprococcus*, are known as probiotics to promote gut health (Louis and Flint, 2009; Vital et al., 2014). A decreased number of *Bifidobacteria* is associated with enhanced gut permeability, systemic inflammatory status, and malnutrition in older adults (Guigoz et al., 2008; Odamaki et al., 2016). Thus, the presence of these bacterial families may favor the anti-inflammatory gut micro-environment.

As mentioned above, most of the changed genera observed in the AD gut flora after NR treatment were linked to inflammatory functions. For instance, *Oscillospira* were reduced in diseases related to inflammation (Gophna et al., 2017). Similarly, *Clostridium* represents 95% of the *Firmicutes* phyla in humans and was found to be less abundant in intestinal inflammatory disorder patients (Rinninella et al., 2019). *Dehalobacterium* decreased in a mouse model exposed to perfluorooctanoic acid, a liver toxicant (Wang et al., 2021). It was also a potential biomarker for diagnosing surgery-induced cognitive dysfunction (Zhan et al., 2019). *Butyricicoccus* has an anti-inflammatory effect (Eeckhaut et al., 2013; Wang et al., 2019; Chang et al., 2020) and a lower relative abundance of *Butyricicoccus* was associated with the neurobehavioral decline in elderly individuals (Bajaj et al., 2016). Additionally, the bacterial species of *Butyricicoccus* extended the lifespan of *Caenorhabditis elegans* (Kwon et al., 2018). These altered genera are potential biomarkers associated with AD and 8 weeks of NR treatment normalized their relative abundances in AD mice. Future interventional studies, such as depleting the intestinal microbiota in AD mice or fecal transplantation from healthy WT mice into microbiota-depleted AD mice, are required to decipher the causal relationship between NR metabolism and gut microbiome in AD mice.

In this study, we did not find species harboring previous reported PnuC-like nicotinamide riboside transporter, e.g., *Haemophilus influenzae*, Streptococcus sp., *Actinobacillus spp.*, *Bacillus thuringiensis*, and *Pasteurella spp.* (Sauer et al., 2004; Magnúsdóttir et al., 2015). This may be due to the limitation of using 16S rDNA data to study composition at species level. Here, we only identified 15 bacteria at the species level, and the rest are unclassified. However, at higher taxonomy levels, we found the abundance of pylum *Actinobacteria*, genus *Bifidobacterium*, and genus *Desulfovibrio*, likely relevant to NAD biosynthetic pathways (Van der Meulen et al., 2006; Sorci et al., 2010), were decreased in AD-Veh mice and increased after NR treatment in AD-NR mice. This suggests that the gut microbiome could contribute to NAD^+^-related deficits in AD mice and NR supplementation might be able to ameliorate the phenotypes. However, whether it is mediated by PnuC-like nicotinamide riboside transporter is still unknown. Further metagenomics studies are required.

In conclusion, to our knowledge, this is the first report on the effects of NR supplementation on gut microbiota in an AD mouse model. We found lower microbiota diversity in AD mice and identified several bacterial markers related to gut dysbiosis and inflammation in AD mice. Disrupting gut microbiome homeostasis in AD mice may contribute to AD pathology. Notably, NR treatment increased the microbiota diversity and restored some probiotic bacteria that are beneficial to gut-brain communication and anti-inflammatory functions, which may contribute to the effect of NR on alleviating AD symptoms. From the relevant research on NR and AD from mice, we might expect that supplementation of NR at a relatively early stage of AD may ameliorate some AD features, including cognitive dysfunction and microbiome aspects. Therefore, we were to speculate if NR supplementation is started in the early stage of the disease, it may have the potential to alleviate or inhibit the occurrence and progression of AD.

## Data availability statement

The datasets presented in this study can be found in online repositories. The names of the repository/repositories and accession number(s) can be found in the article.

## Ethics statement

The animal study was reviewed and approved by National Institute on Aging.

## Author contributions

YH, QM, DC, and VB conceived and designed the study. XC and QM performed the data acquisition, analysis, and interpretation. YH and YW contributed to mice treatment and fecal DNA extraction. QM performed the microbial community sequencing. DC, SD, and KB provided suggestions and critical revisions to the manuscript. XC and VB drafted the manuscript. All authors contributed to editing and proofreading of the final draft, contributed to the article, and approved the submitted version.

## Footnotes

1
https://insights.envigo.com/hubfs/resources/data-sheets/2018sx-datasheet-0915.pdf

2
https://cran.r-project.org/web/packages/vegan/index.html

3
https://cran.r-project.org/web/packages/cluster/index.html

4
https://www.microbiomeanalyst.ca/MicrobiomeAnalyst/home.xhtml

## Abbreviations

AD mice or 2xTg AD mice: APP/PS1 double transgenic mice
Aβ: amyloid beta
ACE: abundance-based coverage estimator
AD: Alzheimer’s disease
ASD: autism spectrum disorder
LEfSE: linear discriminant analysis effect size
NAD: nicotinamide adenine dinucleotide
NR: nicotinamide riboside
MS: multiple sclerosis
OTUs: operation taxonomic units
PCoA: principal coordinate analysis
PD: Parkinson’s disease
rRNA: ribosomal RNA
SCFA: short chain fatty acids
WT: wild-type.
