# Supplementary material for: Nicotinamide adenine dinucleotide supplementation drives gut microbiota variation in Alzheimer’s mouse model
Source: Front Aging Neurosci. 2022 Sep 15;14:993615. doi: 10.3389/fnagi.2022.993615 (PMC9520302; doi:10.3389/fnagi.2022.993615)
Supplement: Supplementary file 1 [file Presentation_1.pdf]

Supplementary Material

1 Supplementary Data

The original sequencing data have been uploaded to Sequence Read Archive (SRA). The link is https://dataview.ncbi.nlm.nih.gov/object/PRJNA853566? reviewer=n89967ja37m6q34um1vvtr371j. Further inquiries can be directed to the corresponding author/s.

1.1 Supplementary Figures

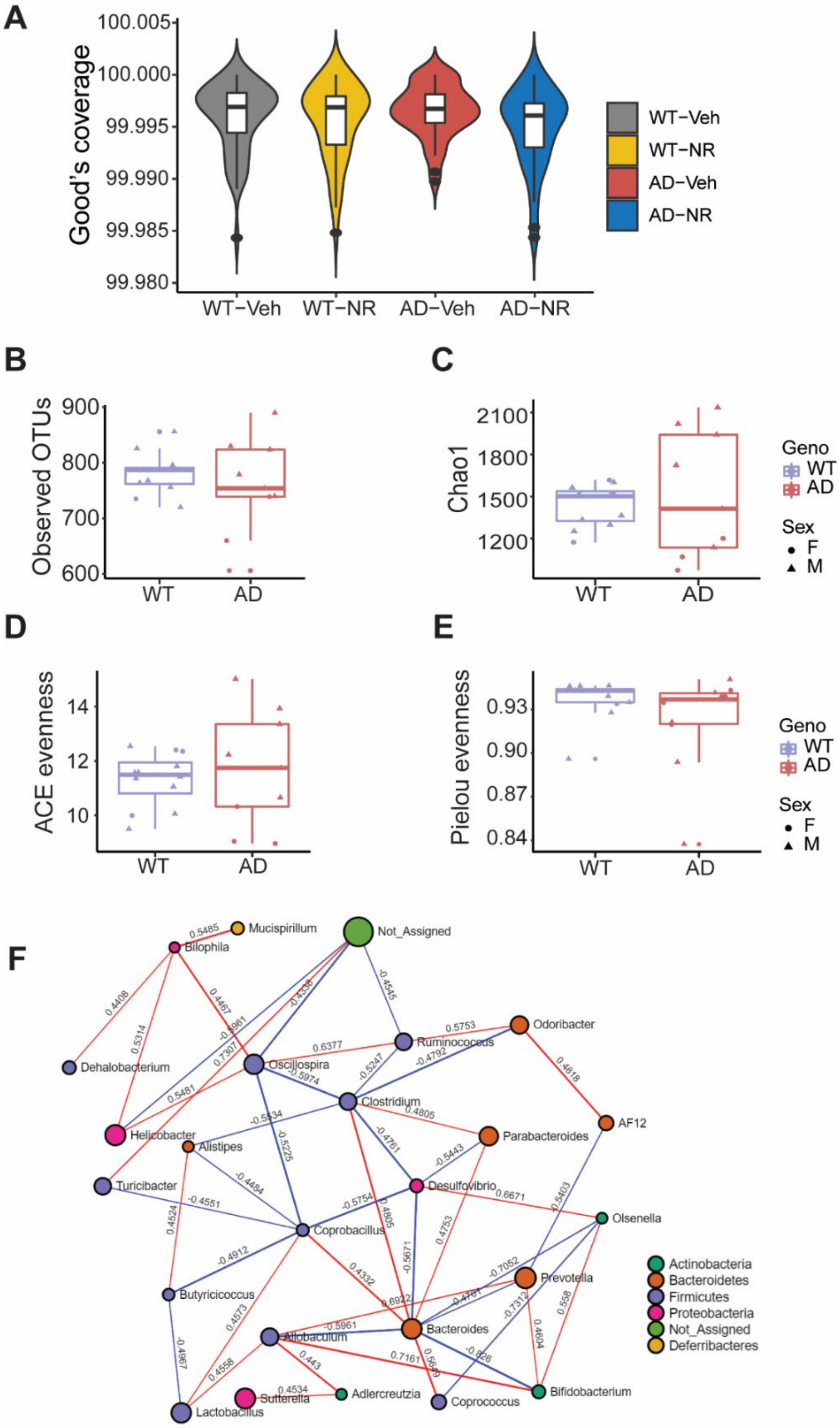

Supplementary Figure 1. The unchanged microbiome diversity measurements and correlation network at the genus levels. **(A)** Average Good's coverage estimates (%) represent the mean ± SD of each group. **(B-C)** Microbiome richness was measured by Observed OTUs **(B)** and Chao1 **(C)**, and there was no difference between AD and WT mice. Evenness was measured by ACE evenness **(D)** and Pielou evenness **(E)** and showed no difference between AD and WT mice. **(F)** The correlation network was analyzed by Spearman rank correlation at the genus level. The WT/AD microbial dysbiosis index is 4e-04, showing the imbalance of microbial community between WT and AD mice. P Values were corrected for multiple testing with Benjamini-Hochberg false discovery rate correction (q value). The colors of each node indicate the phylum it belongs to. The orange lines show positive correlations, and the blue lines indicate negative correlations. the size is based on the number of connections to the taxon. AD (n = 9, 6 males and 3 females, 12-month-old) versus WT (n = 11, 9 males and 3 females, 12-month-old).

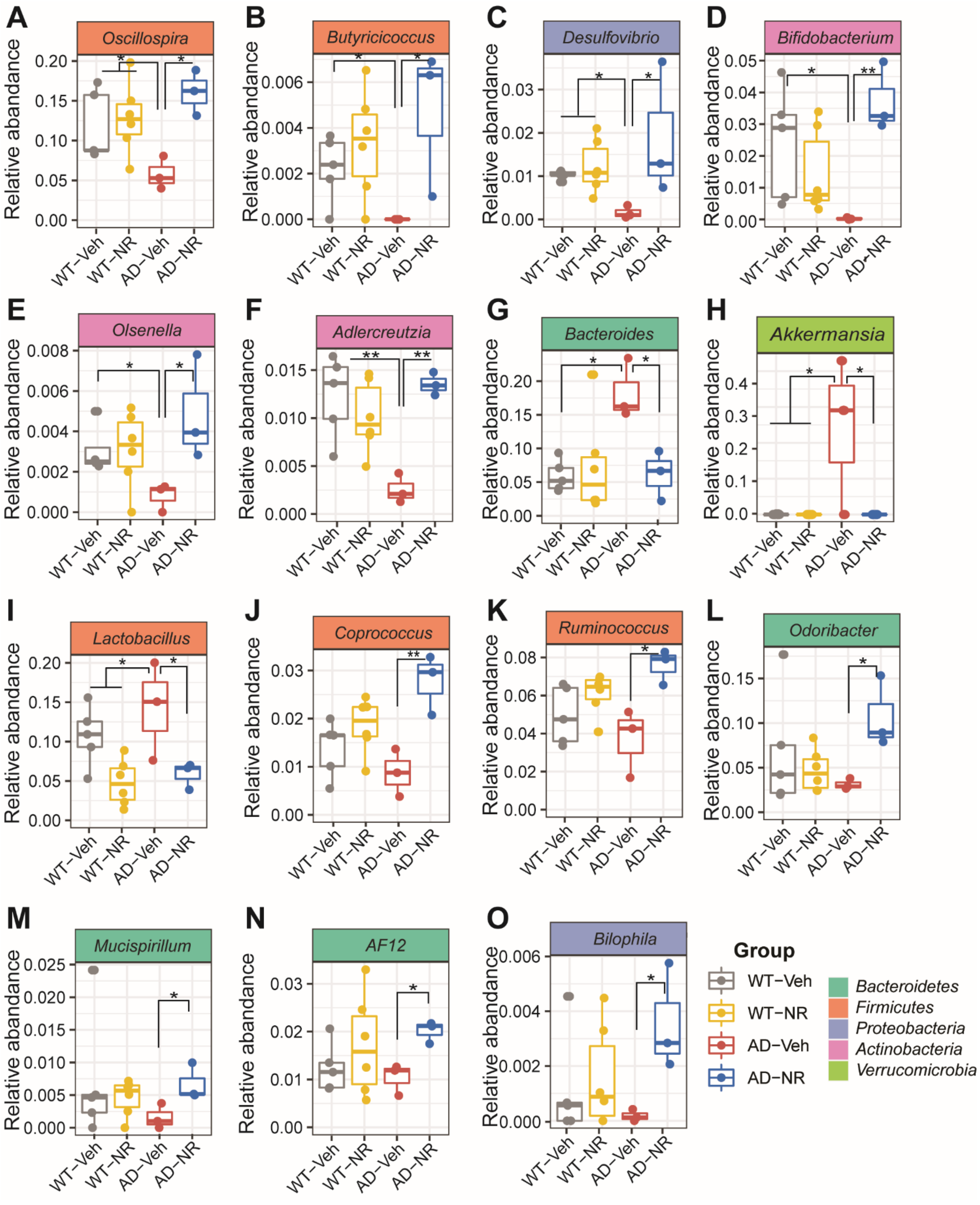

Supplementary Figure 2. Represented genera changed after NR treatment in AD mice compared to controls. (A-F) The relative abundance of genera *Oscillospira* (A), *Butyricicoccus* (B), *Desulfovilbrio* (C), *Bifidobacterium* (D), *Olsenella* (E) and *Adlercreutzia* (F) decreased in AD mice and increased

after eight weeks of NR treatment. (G-I) The relative abundance of genera *Bacteroides* (G), *Akkermansia* (H) and *Lactobacillus* (I) increased in AD mice and decreased after eight weeks of NR treatment. (J-O) The relative abundance of genera *Coprococcus* (J), *Ruminococcus* (K), *Odoribacter* (L), *Mucispirillum* (M), *AF-12* (N) and *Bilophila* (O) only increased in AD mice after NR treatment. Data are shown as all samples with a median value, IQR and participant data points. *P < 0.05, **P <0.001, two-way ANOVA followed by Tukey's multiple comparisons. WT-Veh (4 males and 1 female), WT-NR (4 males and 2 females), AD-Veh (2 males and 1 female), AD-NR (2 males and 1 female), 12-month-old.
